# Tumor-Intrinsic or Drug-Induced Immunogenicity Dictates the Therapeutic Success of the PD1/PDL Axis Blockade

**DOI:** 10.3390/cells9040940

**Published:** 2020-04-10

**Authors:** Alessandra Rossi, Valeria Lucarini, Iole Macchia, Paola Sestili, Carla Buccione, Simona Donati, Maria Ciccolella, Antonella Sistigu, Maria Teresa D’Urso, Anna Maria Pacca, Enrico Cardarelli, Fabrizio Mattei, Enrico Proietti, Giovanna Schiavoni, Laura Bracci

**Affiliations:** 1 Department of Oncology and Molecular Medicine, Istituto Superiore di Sanità, 00161 Rome, Italy; alessandra.rossi@uniroma1.it (A.R.); valeria.lucarini@opbg.net (V.L.); iole.macchia@iss.it (I.M.); paola.sestili@iss.it (P.S.); carla.buccione@gmail.com (C.B.); simona.donati@iss.it (S.D.); mariaciccolella06@gmail.com (M.C.); fabrizio.mattei@iss.it (F.M.); enrico.proietti@iss.it (E.P.); 2 Department of Research, Advanced Diagnostics and Technological Innovation, IRCCS - Regina Elena National Cancer Institute, 00144 Rome, Italy; antonella.sistigu@gmail.com; 3 Istituto di Patologia Generale, Università Cattolica del Sacro Cuore, 00168 Rome, Italy; 4 Animal Research and Welfare Centre, Istituto Superiore di Sanità, 00161 Rome, Italy; mariateresa.durso@iss.it (M.T.D.); annamaria.pacca@iss.it (A.M.P.); enrico.cardarelli@iss.it (E.C.)

**Keywords:** programmed death ligand, chemotherapy, mouse models, chemo-immunotherapy, immune response, myeloid infiltrate, memory subsets

## Abstract

Immunotherapy with immune checkpoint inhibitors (ICIs) has revolutionized cancer treatment providing unprecedented clinical benefits. However, many patients do not respond to ICIs as monotherapy or develop resistance. Combining ICI-based immunotherapy with chemotherapy is a promising strategy to increase response rates, but few rationale-driven chemo-immunotherapy combinations have reached the clinical arena thus far. In the present study, we show that combined anti-PDL1 and anti-PDL2 antibodies optimally synergize with cyclophosphamide but not with cisplatin, and that the magnitude and duration of the therapeutic response is dependent on the immunogenic potential of the drug and of the tumor itself. Hallmarks of successful therapeutic outcomes were the enhanced infiltration by myeloid (mainly cross-presenting dendritic cells, eosinophils, and monocytic myeloid cells) and T lymphocytes into the tumor tissue and the expansion of circulating memory pools. Overall, our results suggest that immunomodulating chemotherapy can be exploited to increase the efficacy of PD1/PDL axis inhibitors in vivo, and that the magnitude of the synergic therapeutic response is affected by tumor-intrinsic immunogenicity.

## 1. Introduction

Immune checkpoints include membrane-bound and soluble factors that in physiological conditions attenuate T cell effector functions thus preserving peripheral tolerance and avoiding autoimmunity. Besides their physiological role, these inhibitory pathways have been involved in tumor escape from immunosurveillance, a major determinant of disease progression. Indeed, unlike co-stimulatory molecules, inhibitory ligands and receptors are commonly overexpressed in the tumor microenvironment (TME) as compared to healthy tissues, both by tumor and stromal cells [1]. 

The introduction into clinical practice of monoclonal antibodies (Abs) targeting those inhibitory molecules, the immune checkpoint inhibitors (ICIs), has provided unprecedented benefit for cancer therapy in the past decade. The ICIs currently approved for cancer treatment targeting programmed death-1 (PD-1) and its ligand programmed death ligand (PDL)-1 have proven therapeutic efficacy for treatment of advanced melanoma, renal cell cancer, colorectal cancer, and non-small-cell lung cancer (NSCLC) [2,3,4]. Programmed death-1 is expressed by activated T lymphocytes and, upon binding to its ligands, PDL1 and PDL2, results in downregulation of effector functions and cell death of T lymphocytes. Programmed death ligand 1 is constitutively present on both hematopoietic and non-hematopoietic cells and is further regulated by external stimuli. Programmed death ligand 2 expression is inducible on the surface of macrophages, dendritic cells, mast cells, and certain B cell populations [5,6]. By releasing these molecular brakes, ICIs can thus reinstate the anticancer adaptive immune responses [1]. Although PDL1 has been shown to play a prominent role in cancer, PDL2 has been less studied and may have been neglected as a potential target in tumor immunity.

Despite the clinical success, approximately 60–70% of patients do not respond to ICIs as monotherapy [7] with mechanisms that have not been fully elucidated yet. Primary resistance to ICIs has been attributed to intrinsic properties of some tumors such as a low mutational burden [7,8,9], defective antigen presentation [10], and limited tumor infiltration [11]. Mechanism of acquired resistance have also been reported, including IFNγ-induced upregulation of PDL1 [1,12] and TCR-dependent upregulation of additional exhaustion markers on T cells such as T lymphocytes, including T-cell immunoglobulin and mucin-domain containing-3 (Tim-3), and lymphocyte activation gene 3 (Lag-3) [13]. Recently, sustained interferon (IFN)-αβ signaling has also been associated to secondary resistance to anti-PD1 therapy [14].

Combining ICI-based immunotherapy with chemotherapy or targeted therapies has been proposed as a promising strategy to increase response rates. Combinations of anti-PD1 Abs with chemotherapy, mainly tested in NSCLC, resulted in good outcomes but still with average response rates in no more than 50–60% of treated patients [7]. The rationale behind chemo-immunotherapy combinations is based on the so called “off target” effects of chemotherapy, referring to immunomodulatory effects of many common drugs that contribute to their therapeutic efficacy including promotion of tumor specific immune responses [15]. In the majority of the abovementioned studies, chemotherapy and immunotherapy were administered concurrently, disregarding the complex interplay between the two treatments that, indeed, still needs to be fully elucidated to allow the design of optimal combination strategies [7]. 

We have here tested the therapeutic efficacy of monoclonal Abs targeting PDL1 and PDL2 molecules in combination with cyclophosphamide (CTX), an alkylating agent endowed with well-characterized immunomodulatory effects [15,16,17], in mouse models of lymphoma (EG.7-OVA) and fibrosarcoma (MCA205) with distinct intrinsic immunogenic potential. We show that an optimal therapeutic efficacy is achieved when CTX treatment is followed by administration of both anti-PDL1 and anti-PDL2 Abs with the former playing a major role. In a less immunogenic tumor model, the combination with immunogenic chemotherapy can substantially increase the therapeutic efficacy of PDL blockade as compared to non-immunogenic chemotherapy. Overall, our results suggest that immunomodulating chemotherapy can be exploited to prime the TME to immune checkpoint inhibition, for instance, by increasing the availability of tumor antigens upon cell death and by subverting the proportion of cytotoxic versus immunosuppressive cells thus paving the way for optimal action of PD1/PDL axis inhibitors. Nevertheless, the intrinsic immunogenicity of tumors may play a role in shaping the quality of antitumor immune responses affecting the magnitude and duration of the immune response elicited.

## 2. Materials and Methods

### 2.1. Mice

Six-to-seven-week-old C57Bl/6 (H-2^b^) female mice were purchased from Charles River Laboratories (Calco, Italy) and housed in the animal facility at Istituto Superiore di Sanità according to the current Italian law guidelines (D.Lgs.vo 26/14). 

### 2.2. Cell lines and Reagents

The EG.7-OVA thymoma (ATCC^®^ CRL-2113™), MCA205 fibrosarcoma cells and MCA205-*Tlr3^−/−^* obtained from mice lacking *Tlr-3* (kindly provided by Zitvogel, Gustave Roussy Cancer Campus, Villejuif, France), were cultured in RPMI 1640 supplemented with 10% heat-inactivated fetal bovine serum (FBS, Lonza), 2 mM L-Glutamine (Lonza), 0.1 U/mL penicillin, 0.1 mg/mL streptomycin (Lonza), 10 mM HEPES, 1.0 mM sodium pyruvate (NaPir), and 0.05 mM β-mercaptoethanol (β-ME) (all from Lonza), hereafter referred to as complete RPMI, and split every three days. Gentamicin (G-418 sulphate, Gibco, 0.4 mg/mL) was added to EG.7-OVA at every medium change. The cell lines were routinely tested for the absence of mycoplasma and passaged for no more than four times from thawing. Cyclophosphamide (CTX, Sigma–Aldrich, St. Louis, MO, USA), the in vitro active analogue of CTX mafosfamide (4-thioethane sulfonic acid salt of 4-hydroxy-cyclophosphamide, MAFO, Sigma) and cisplatin (cis-diamminedichloroplatinum (II), CDDP, Sigma) were dissolved in saline and filtered sterile before use. Type I Interferon (IFN-I) was produced at the department of Oncology and Molecular Medicine as previously described [16]. A mock preparation was used as specificity control.

### 2.3. Primary Cells

Leukocytes from blood and spleen were collected as previously described [18]. Briefly, blood was collected from the retrorbital plexus, placed in EDTA-coated 1 mL tubes and centrifuged. Plasma was removed and blood cells were diluted in ACK lysing buffer (150 mM NH_4_Cl + 10mM KHCO_3_ + 0.1 mM Na_2_EDTA, pH 7.2–7.4) for erythrocyte lysis. Samples were centrifuged in complete RPMI 1640 to neutralize the ACK buffer activity, resuspended in complete RPMI, and counted in trypan blue 0.4% solution.

Spleens and tumor-draining lymph nodes (LNs) were surgically removed from euthanized mice, placed onto a cell strainer (70–100 μm pore size), laid on a sterile Petri dish containing ACK lysing buffer, and gently pressed with the plunger of a sterile syringe to grind the tissue. Complete RPMI was added to block lysis and cells were centrifuged before counting in trypan blue 0.4% solution.

Tumors were surgically removed from euthanized mice and cut into small pieces with sterile scissors before incubation with 1 mg/mL Collagenase Type and 325 KU/mL DNAse for 30 min at 37 °C as previously described [16]. The digested material was filtered by a 70 μm cell strainer and centrifuged before counting in trypan blue 0.4% solution. 

Dendritic cells (DC) were generated from murine bone marrow as previously described [19]. Briefly, erythrocyte-depleted bone marrow cells flushed from the femurs and tibiae of C57BL/6 mice were cultured at 1 × 10^6^ cells/mL in complete Dulbecco medium (IMDM) (Lonza) containing 10% FCS, 50 μM β-ME, 100 U/mL penicillin, 100 μg/mL streptomycin, 100 U/mL polymyxin B, and 10 ng/mL recombinant murine granulocyte-macrophage colony-stimulating factor (rmGM-CSF) (R&D Systems, Abingdon, Oxon, United Kingdom). Fresh medium was added every other day. On day 6, loosely adherent cells were harvested, washed, and replated in fresh medium. Phenotypic analysis and functional assays were performed between days 10 and 14. The CD11c^+^ cells ranged between 95% and 98% without any further sorting or treatment.

### 2.4. In Vitro Treatments

To analyze PDL expression by tumor cells, EG.7-OVA or MCA205 (*Tlr-3^+/+^* and *Tlr-3^−/−^*) were cultured for 24 h in complete RPMI with mafosfamide (MAFO, (10 µM), the in vitro active metabolite of CTX, or with cisplatin (cis-diamminedichloroplatinum (II), CDDP, 6 µM). In some experiments, anti-IFNAR1 Ab (MAR1-5A3) or Control IgG1 were added. To evaluate the immune-stimulating effects of chemotherapy-induced tumor cell death, 10^6^ MCA205 fibrosarcoma cells were plated in complete RPMI containing MAFO (28 µM) or CDDP (150 µM) and were incubated for 4 h at 37 °C, 5% CO_2_. Cells were then harvested, extensively washed to remove the drugs, resuspended in 0.1 mL of PBS, and injected peritumorally in tumor-bearing mice. In some cases, chemotherapy-treated tumor cells were cocultured with bone marrow-derived DC at a 2:1 ratio for additional 20 h. Some DC coltures were stimulated with 100 ng/mL of Lipopolysaccharide (LPS, Sigma-Aldrich, St. Louis, Missouri, USA) as maturation stimuli (positive control). Cell cultures were then stained with fluorescence-labeled Abs against CD11c (N418), MHC-II (M5/114), CD86 (PO3.3), CD80 (16-10A1) (Miltenyi Biotech) and analyzed by flow cytometry. Percentages and mean fluorescence intensities (MFI) of cells expressing the activation markers were recorded.

### 2.5. Therapeutic Protocol

C57Bl/6 female mice were injected subcutaneously (s.c.) with 5 × 10^6^ EG.7-OVA or with 8 × 10^5^ MCA205-*Tlr3^+/+^* or with the same dose of MCA205-*Tlr3^-/-^* in Matrigel (0.1 mL/mouse) (BD Biosciences). When tumors reached a mean diameter of 9 ± 2 mm, they were treated intraperitoneally (i.p.) with 100 mg/kg CTX or 2.5 mg/kg CDDP followed by 3 injections of anti-PDL1 (clone 10F.9G2) and/or anti-PDL2 (clone TY25) Abs (InVivoMAb, BioXcell) in dilution buffer (InVivoPure pH 6.5, BioXcell). The first injection (150 μg/mouse) was given s.c. peritumorally 3 days after chemotherapy, the subsequent injections (250 μg/mouse) were given i.p. on days 7 and 10 after chemotherapy. In some experiments, mice received one s.c. peritumoral injection of 1000U IFN-I or mock instead of chemotherapy followed by three anti-PDL1/2 Ab administrations as detailed above. Control groups received the same volume of saline instead of the drugs and of control isotypes (IgG2b and IgG2a, InVivoMAb, BioXcell), instead of the specific Abs. Tumor growth was measured by a caliper twice a week. In some experiments, long-term survivors were challenged with 10^6^ live EG.7-OVA cells s.c. into the right flank and the development of a new tumor mass was monitored twice a week and measured with a caliper. 

### 2.6. IFNγ ELISpot

For ELISpot assay, 10^5^ blood leukocytes were added to each well in triplicate in pre-coated PVDF-96 well plates and cultured in complete RPMI in the presence of 10^4^ irradiated (20Gy) tumor cells, OVA_257–264_ peptide (SIINFEKL, 10 µg/mL, Invitrogen) or Concanavalin-A (Con-A, 5 μg/mL) as previously described [18]. The spot number was counted by using an ELISpot reader (Aelvis) and expressed as the number of spot-forming cells (SFC)/10^5^ cells.

### 2.7. Flow Cytometry

Cells from spleens or LN (2 × 10^5^) and tumors (10^6^) were seeded in 96 well U-bottomed plates, centrifuged twice in staining buffer (PBS + 1% FBS+EDTA 2 mM) and incubated with a fixable cell viability dye (LIVE/DEAD™ Fixable Near-IR -NiR, Dead Cell Stain Kit, ThermoFisher Scientific, Waltham, MA, USA) followed by incubation with full FBS to saturate non-specific Ab binding sites. The following fluorescent Abs appropriately diluted in staining buffer were added to cell samples in different combinations: CD19 (MB19-1), CD3 (17A2), CD8 (53–6.7), CD4 (GK1.5), CD11b (M1/70), CD11c, F4/80 (BM8), Ly6C (HK1.4), Ly6G (1A8), PDL1 (10F.9G2), PDL2 (MIH37), PD-1 (29F.1A12), Siglec-F (E50–2440), MHC-I (AF6–88.5) (all from Biolegend), MHC-II (M5/114), CD86 (PO3.3), CD80 (16–10A1), CD103 (2E7). Biotinylated Abs were detected by streptavidin BV421 (ThermoFisher Scientific, Waltham, MA, USA). Cells were resuspended in paraformaldehyde 1% and kept at + 4 °C in the dark until acquisition on a 4 lasers flow cytometer (Gallios, Beckman Coulter, Miami, FL, USA). Data analysis was performed by using Kaluza software (Beckman Coulter, Miami, FL, USA). Immune cell populations were defined as follows: CD45^+^CD3^+^CD19^−^CD4^+^CD8^−^ (CD4^+^ T cells); CD45^+^CD3^+^CD19^−^CD4^−^CD8^+^ (CD8^+^ T cells); CD45^+^CD3^+^CD8^+^/CD4^+^CD44^hi^CD62L^hi^ (T central memory); CD45^+^CD3^+^CD8^+^/CD4^+^CD44^hi^CD62L^lo^ (T effector memory); CD45^+^CD3^+^CD8^+^/CD4^+^CD44^+^CD62L^-^ (T effector); CD45^+^CD3^+^CD8^+^/CD4^+^CD44^-^CD62L^hi^ (T naïve); CD45^+^CD3^−^CD19^+^ (B cells); CD45^+^CD3^+^CD4^+^CD25^+^Foxp3^+^ (regulatory T cells); CD45^+^CD11b^hi^MHC-II^-^Siglec-F^hi^Ly6G^−^ (eosinophils); CD45^+^CD11b^+^Ly6C^hi^Ly6G^−^ (Mo-MDSC); CD45^+^CD11b^+^Ly6C^lo^Ly6G^+^ (PMN-MDSC); CD45^+^CD11b^+^MHC-II^+^F4/80^+^ (macrophages); CD45^+^CD11c^+^MHC-II^+^ (DC). For the identification of tumor-infiltrating lymphocytes in EG.7-OVA tumors, cells were gated for morphological parameters (FSC^low^SSC^low^) before analyzing the specific linage markers.

### 2.8. Intracellular Staining

Blood leukocytes were seeded in 96-well U-bottomed plates in complete RPMI medium in the presence of 0.7 µg/mL Brefeldin A (Biolegend), 1 µg/mL Monensin (Biolegend) and anti-CD107a mAb (LAMP-1) and stimulated with 10 μg/mL OVA_257–264_ peptide or with 2 µg/mL Ionomycin (Sigma) plus 0.2 µg/mL phorbol-12-myristate-13-acetate (PMA, Sigma) for 5 h at 37 °C. At the end of incubation, cells were surface stained with fluorescent anti-CD3, anti-CD8, anti-CD44 (IM7), anti-CD62L (MEL-14), anti-PD-1, and Tim-3 (RMT3-23) mAbs (all from Biolegend). Cells were then permeabilized (Fixation/Permeabilization Concentrate, ThermoFisher Scientific) and stained with anti-IFNγ mAb (XMG1.2) or isotype control (Biolegend). In some experiments, to identify Tregs, spleen or tumor cell suspensions were surface stained with anti-CD4, anti-CD25 (PC61) and anti-GITR (DTA-1) mAbs, then permeabilized and stained with anti-Foxp3 mAb (FJK-16s) or matched isotype control (ThermoFisher Scientific, Waltham, MA, USA). Cells were then fixed with 1% paraformaldehyde and stored at 4 °C in the dark until acquisition by Gallios flow cytometer.

### 2.9. mRNA Extraction and Real-Time PCR

Total RNA was extracted from MCA205-*Tlr-3^+/+^* or *Tlr-3^-/-^* and from EG.7-OVA tumor lesions by using TRIsure reagent (Bioline, London, UK). The mRNA was reverse transcribed by means of Tetro cDNA Synthesis Kit (Bioline). Quantitative reverse transcription-PCR (qPCR) with forward and reverse primers (see Table 1 for sequence) for TLR-3 (MCA205 only) and IFNγ, IL-2, IL-4, IL-13, IL-17, IL-33, perforin, granzyme B and HPRT (Eurofins Genomics, Ebersberg, Germany) [20] was performed using Sensimix Plus SYBR Kit containing the fluorescent dye SYBR Green (Bioline) and by means of an ABI 7500 Real-Time PCR system (Applied Biosystems, Thermo Fisher Scientific, Waltham, Ma, USA). Triplicates were performed for each experimental point. Data were normalized to HPRT (2-ΔCt method) and presented as fold change expression versus. control. A Heatmap of the gene expression was generated using R software [21] and its Pheatmap package [22]. 

### 2.10. Morphometric Analysis of MCA205 Tumor Cells

Images from cultured MCA205 cells without (control) or with CDDP or MAFO were acquired by using an EVOS-FL microscopy system and were then thresholded using the Default algorithm of Imagej Software [23]. The thresholded masks were then processed by using the Particle Analysis plugin of ImageJ. Morphometric parameters such as Feret’s diameter, circularity, area and perimeter, of each cell [24] were calculated for a representative micrograph per each experimental condition.

### 2.11. Statistical Analysis

One-way ANOVA analysis of variance was performed to compare means among multiple groups, followed by post hoc testing (Tukey). The Mann–Whitney test was used for the non-parametric analysis of differences between two groups. The Log-rank Mantel–Cox test was used for the analysis of survival curves. Statistical analysis of gene expression was done with one-way ANOVA plus the Newman–Keuls post-hoc test. Values were considered significant when the *p*-value was below 0.05.

## 3. Results

### 3.1. PD1-PDL Axis Blockade Induces Systemic Polyclonal Immune Activation but no Tumor Specific Immune Responses or CD8+ T Cells and Mice Survival

**Figure 1:**
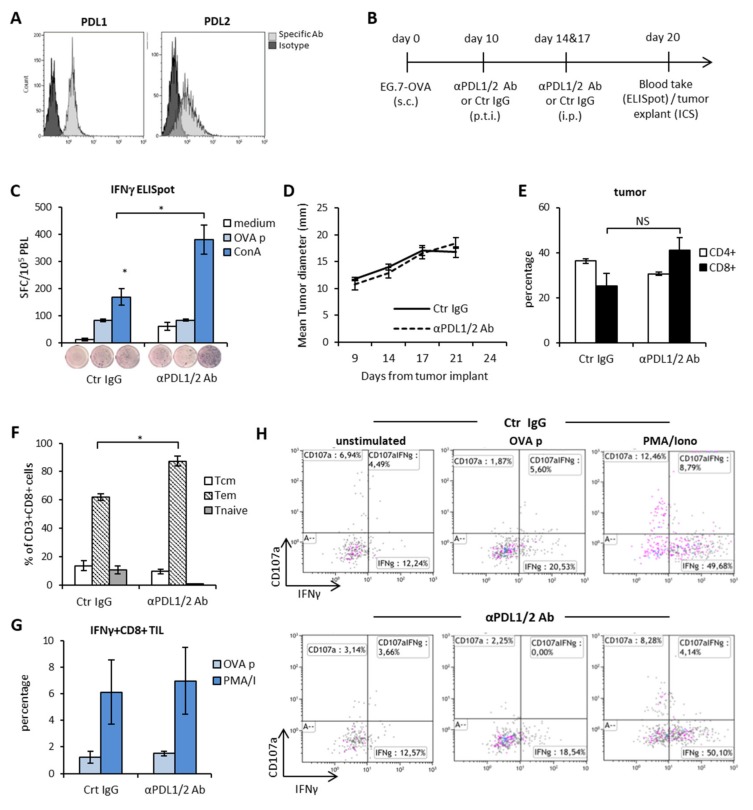
The PD1-PDL axis blockade enables systemic immune activation, but poor tumor-specific reactivity and therapeutic outcome in a highly immunogenic tumor model. (**A**) Expression of PDL1 and PDL2 molecules on EG.7-OVA cells in vitro. (**B**) Schematic representation of the experimental design. (**C**) IFNγ-ELISpot on blood leukocytes (PBL) collected 3 days after the last injection with anti-PDL1 + anti-PDL2 Abs or Control IgG (Crt IgG) and incubated o.n. with the indicated stimuli. Data are represented as mean ± SEM. Representative wells from each condition are shown. (*n* = 5) (**D**) Mean tumor size of mice treated with anti-PDL1 + anti-PDL2 Abs or Crt IgG (*n* = 5). (**E**) Percentage of tumor-infiltrating CD3^+^CD4^+^ and CD3^+^CD8^+^ T lymphocytes (TIL) 3 days after the last Ab injection. Data are expressed as mean ± SEM (*n* = 5). (**F**) Effector memory (Tem) versus central memory (Tcm) CD8^+^ phenotypes in the tumor bed 3 days after the last Ab injection (*n* = 3). (**G**) Percentage of IFNγ^+^ CD3^+^CD8^+^ TIL assessed by ICS upon stimulation with OVA peptide (OVA p) or PMA/Ionomycin or medium (unstimulated). Data are expressed as mean fold increase as compared to unstimulated samples (*n* = 3). (H) Representative dot plots of IFNγ and/or CD107a staining in CD3^+^CD8^+^ gated TIL. * *p* < 0.05. NS = non-significant.

Immunogenic tumors are recognized as the ideal candidates for ICI therapy [25]. Due to the constitutive expression of peptides from the chicken egg albumin (Ovalbumin, OVA) bound to MHC class I molecules and to the surface expression of PDL1 and PDL2 molecules (Figure 1A), EG.7-OVA thymoma is considered an immunogenic tumor model. To investigate the rate of success of double PDL1 and PDL2 blockade in immunogenic tumors, C57Bl/6 female bearing EG.7-OVA tumors were treated with anti-PDL1 and anti-PDL2 blocking Abs or control IgG diluted in saline as control (Figure 1B) every three/four days starting at day 13 from tumor implant. Three days after the last Ab injection, we measured the frequency of tumor-specific effector CD8^+^ T cells in the peripheral blood (PBL) as readout of antitumor response induced by the immunotherapy. As shown in Figure 1C, the rate of IFNγ-secreting cells in response to stimulation with the tumor antigen (OVA peptide) was similar between the two groups of treatment. Instead, release of IFNγ in response to stimulation with the mitogen Con-A was more pronounced in mice treated with anti-PDL1/2 Abs as compared to controls. Also, the spontaneous release of IFNγ from blood leucocytes was superior in mice treated with anti-PDL1/2 with respect to controls. This observation is corroborated by the lack of any therapeutic effect by anti-PDL1/2 therapy both in terms of tumor size (Figure 1D) and of mice survival (not shown). These data suggest that blocking PD1/PDL signaling in vivo augments T cell activation and expansion but has no impact on tumor-specific immunity. From the analysis of the phenotype and function of tumor-infiltrating lymphocytes (TILs), we observed that the frequency of CD4^+^ and CD8^+^ T cells in the two treatment groups was similar (Figure 1E), while anti-PDL treatment was associated with higher frequencies of CD8^+^ T cells with an effector-memory phenotype (CD44^hi^CD62L^-^, Figure 1F). However, when we analyzed the ability of these cells to release IFNγ in response to antigen-specific stimulation, again we observed no difference between the two groups (Figure 1G, H). Based on these results, we concluded that the PD1/PDLs axis blockade as a monotherapy enhances the functionality of cytotoxic lymphocytes tout-court but has poor impact on tumor-specific immune responses and therapeutic outcome.

### 3.2. Modulation of PD1/PDL Molecules in Tumor and Spleen of Mice Treated with CTX

**Figure 2:**
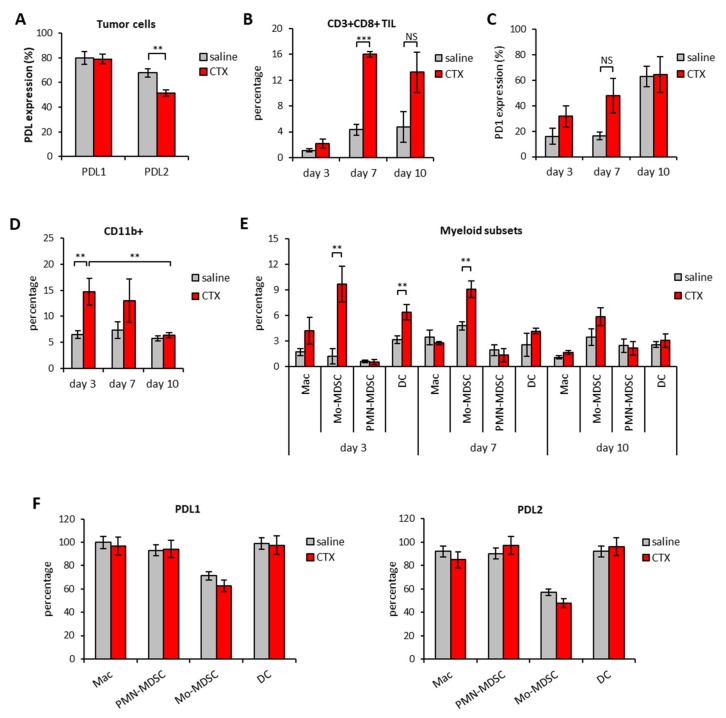
Modulation of PD1/PDL molecules in the tumor of mice treated with CTX. C57Bl/6 mice were treated with 100 mg/kg of CTX or with saline as control (*n* = 5). Tumor mass were excised 3, 7, and 10 days after treatment and the leucocyte subset composition as well as the expression of PD1, PDL1 and PDL2 molecules was evaluated by multicolor flow cytomtery at each time point. **(A)** Ex-vivo expression of PDL1 and PDL2 on tumor cells 3 days after CTX administration (*n* = 5). **(B)** Percentage of CD3^+^CD8^+^ TIL and **(C)** PD1 expression at different time points from CTX or saline injection (*n* = 5). Expression was analyzed after gating on viable FSC^low^SSC^low^ cells. **(D)** Percentage of CD11b^+^ cells in tumor masses at the indicated time-points after saline or CTX injection and **(E)** percentage of the indicated myeloid cell subsets in the tumor microenvironment at different time-points from CTX or saline injection (*n* = 5). **(F)** Percentage of PDL1 and PDL2 expression on tumor-infiltrating myeloid subsets on day 3 after CTX or saline injection (*n* = 5). Expression was analyzed after gating on viable cells. All data are expressed as mean values ± SEM. * *p* < 0.05; ** *p* < 0.01; *** *p* < 0.005. NS = non-significant.

Due to the rising interest in combined treatments for cancer and the need for rationale-driven combinations, we investigated whether cyclophosphamide (CTX), an alkylating agent with well characterized immunomodulating properties [15,26] could enhance the therapeutic efficacy of anti-PDL blocking Abs. In order to establish the best timing for combination, the expression of PDL1 and PDL2 molecules was analyzed in the spleen and tumors of mice implanted with EG7-OVA tumors treated with a single injection of CTX (100 mg/kg). Tumor masses and spleens were excised three, seven and ten days after CTX treatment, along with saline-treated tumors as matched controls. We confirmed the expression of PDL1 and PDL2 on tumor cells that stained positive for both markers irrespective of treatment (Figure 2A). 

**Figure 3:**
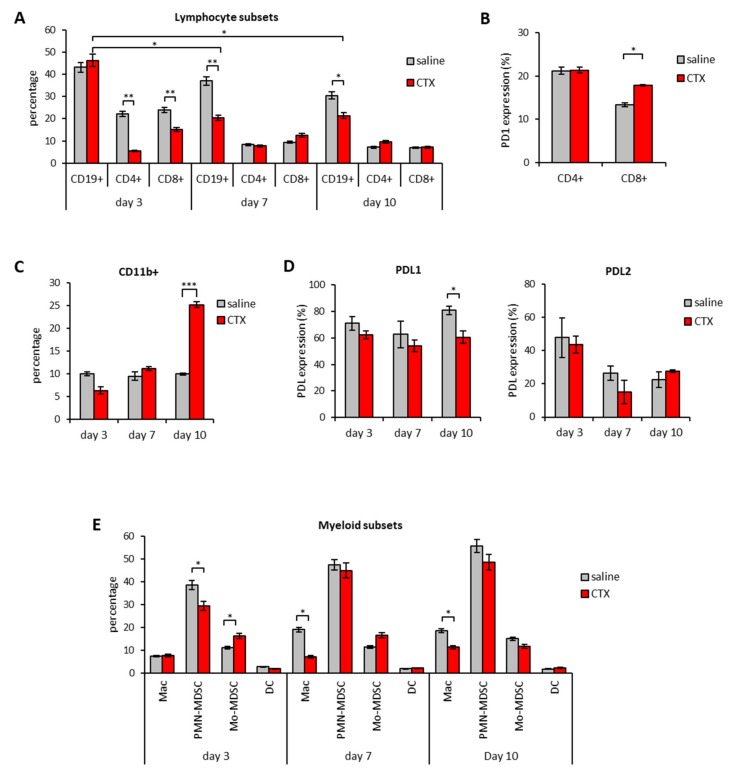
Modulation of PD1/PDL molecules in the spleen of mice treated with CTX. C57Bl/6 mice were treated with 100 mg/kg of CTX or with saline as control (*n* = 5). Three, 7 and 10 days after treatment the leucocyte subset composition as well as the expression of PD1, PDL1 and PDL2 molecules was evaluated in the spleen by multicolor flow cytomtery. **(A)** Percentage of the indicated cell subsets in the spleen of saline or CTX-treated mice (*n* = 5). **(B)** Percentage of PD1 expression in spleen T lymphocytes on day 7 after CTX or saline treatment (*n* = 5). **(C)** Percentage of total myeloid cells and **(D)** of PDL1 and PDL2 expression in the spleen of mice treated with CTX or with saline as control (*n* = 5). **(E)** Relative abundance of the indicated myeloid cell subsets in the spleen at different time-points from CTX or saline injection (*n* = 5). All data are expressed as mean percentage ± SEM. * *p* < 0.05; ** *p* < 0.01; *** *p* < 0.005. NS = non significant.

The injection of CTX induced intratumoral accumulation of CD8^+^ T cells that reached their maximum on day 7 (Figure 2B). The expression of PD1 on CD8^+^ T cells also seemed to increase overtime in the tumor tissue, peaking on day 10 after treatment (Figure 2C). CD4^+^ T cells were also detected in the infiltrate but, unlike CD8^+^, CTX treatment did not change their frequency nor PD1 expression in a statistically significant manner (data not shown). CTX treatment was also associated with increased intratumoral frequencies of CD11b^+^ myeloid cells that peaked on day 3 after treatment (Figure 2D). The detailed analysis of myeloid subsets showed that CTX-treated tumors were enriched in macrophages (Mac), dendritic cells (DC) and monocytic-myeloid derived suppressor cells (Mo-MDSC) as compared to untreated counterparts (Figure 2E). PDL1 and PDL2 were expressed by all myeloid subsets in the TME and their expression was comparable between CTX- and saline-treated groups (Figure 2F). A parallel analysis of spleens from tumor-bearing mice showed that CTX treatment was associated with a substantial decrease in B cells frequency overtime and a short-lived (i.e., day 3) depletion of CD4^+^ and CD8^+^ T cells (Figure 3A). 

Interestingly, a significant increase in the frequency of CD8^+^ PD1^+^ T cells was observed on day 7 after CTX treatment (Figure 3B). CTX also affected the frequency of splenic myeloid cells, which on day 10 after treatment doubled as compared to untreated mice (Figure 3C) and reduced their overall PDL1 expression (Figure 3D). No difference in the expression of PDL1 and PDL2 on CD11b^+^ cells was observed in the other time-points (Figure 3D). From the analysis of the myeloid subset composition, we observed an opposite effect on Mo- and PMN-MDSC on day 3 after treatment and a decrease in Mac frequency at later time-points from CTX administration (Figure 3E).

### 3.3. Therapeutic Synergism between CTX and PDL1/2 Blockade and Immune Correlates of Therapeutic Efficacy

**Figure 4:**
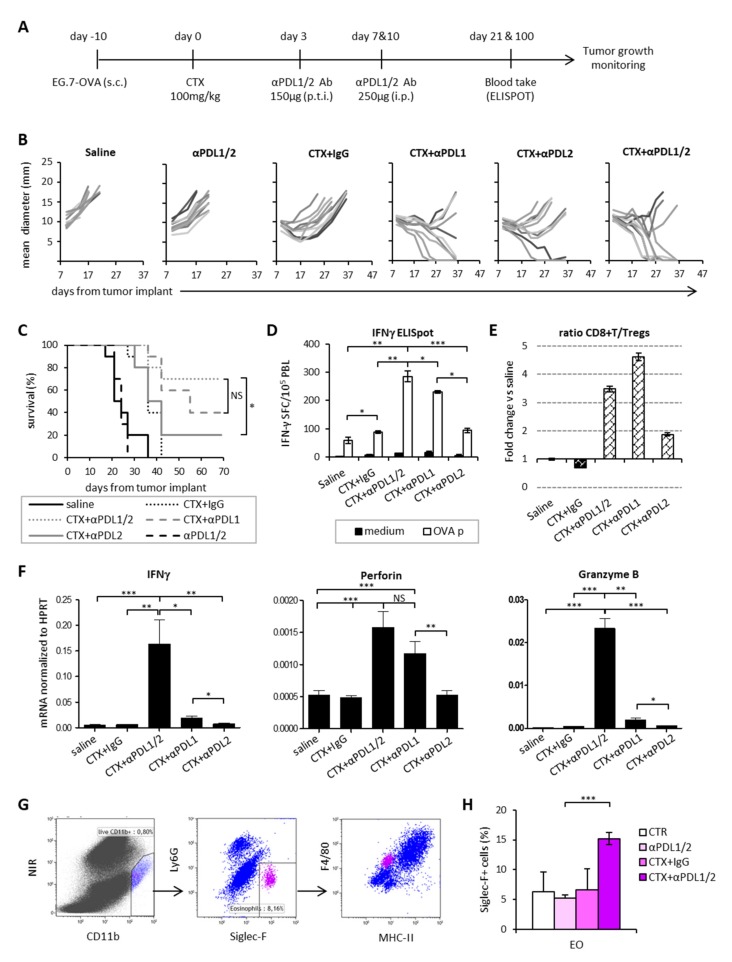
Concomitant PDL1 and PDL2 blockade synergizes with CTX for tumor rejection. **(A)** Schematic representation of the experimental design. **(B)** Mean tumor diameter of individual mice in each experimental group (*n* = 10). **(C)** Percentage of surviving mice in each experimental group (*n* = 10). **(D)** Frequency of IFNγ secreting blood leucocytes (PBL) collected on day 21 from treatment initiation and stimulated with MHC class I-restricted OVA peptide (OVA p) or medium as control (*n* = 10). Data are expressed as mean ± SEM. **(E)** CD8^+^ T/Tregs ratio in tumor microenvironment expressed as fold change versus saline-treated animals (*n* = 5). Tumor masses were explanted on day 14 from treatment initiation and processed as described in Materials and Methods section. Tumor infiltrating lymphocytes (TIL) were plotted after gating on viable FSC^low^SSC^low^CD45^+^ cells. Data are expressed as mean ± SEM. One representative experiment out of two with similar results is shown. **(F)** Expression levels of cytotoxic factors in tumor tissue explanted from mice treated as indicated on day 14 from treatment initiation (*n* = 5). Data are expressed as mean ± SEM after normalization versus HPRT expression. One representative experiment out of two with similar results is shown. **(G)** Gating strategy to identify eosinophils (EO) in the tumor bed of mice treated as indicated 14 days after treatment initiation. **(H)** Percentage of tumor-infiltrating EO after gating on CD11b^+^Ly6G^-^Siglec-F^+^ (*n* = 5). Data are expressed as mean ± SD. * *p* < 0.05; ** *p* < 0.01; *** *p* < 0.005. NS = non-significant.

Based on the information collected from the analysis of leucocyte subsets and on their relative PD1/PDL expression in the course of CTX treatment, we designed a combined chemo-immunotherapy protocol aimed at maximizing the immune-modulating effects of CTX by neutralizing PDL1 and PDL2 inhibitory molecules. To this aim, we treated mice bearing EG.7-OVA tumors with a single dose of CTX (100 mg/kg) followed by a peritumoral injection of anti-PDL1 and anti-PDL2 Abs to block intratumoral PDLs, given three days later (Figure 4A), i.e., at the time of maximum myeloid infiltration into the tumor (Figure 2D). On day 7 and 10 after CTX injection the same Abs were administered intraperitoneally (i.p.) to shield activated splenic PD1^+^CD8^+^ T cells from inhibition (Figure 3B) and to neutralize the inhibitory potential of the expanded CD11b^+^ cells (Figure 3C), respectively. As shown in Figure 4B, either CTX or anti-PDLs given as monotherapy did not induce a significant delay in tumor growth as compared to saline. However, sequential treatment with CTX and either anti-PDL1 Ab or anti-PDL2 Ab induced a reduction in tumor size that was more pronounced when Abs were co-administered (Figure 4B) leading 60% of mice to survival (Figure 4C). Of note, tumor-free mice in each group survived for more than 2 months, suggesting the establishment of a long-lasting antitumor immunity. Twenty-one days after therapy initiation, blood samples were assayed for the frequency of IFNγ-secreting CD8^+^ T cells to find immune correlates of therapeutic response (Figure 4D). Combination of CTX with anti-PDL2 Ab did not significantly affect the antitumor immune response, which, instead, was dramatically enhanced when CTX was administered in combination with anti-PDL1 blockade. Concomitant blockade of both molecules (CTX + anti-PDL1/2) further increased the number of tumor-specific CD8+ T cells producing IFNγ (Figure 4D). Notably, the frequency of circulating IFNγ-producing cells in all groups correlated with therapy outcome in terms of tumor size and mice survival (Figure 4B–D). Blood samples from long-term survivors were assayed again by IFNγ-ELISpot 100 days after treatment to evaluate the persistence of memory responses. Interestingly, survivors from the group treated with CTX and anti-PDL1 + PDL2 Abs displayed higher tumor-specific reactivity as compared to mice treated with CTX + anti-PDL1 Ab (Supplementary Materials Figure S1A). When subjected to challenge with live tumor cells, 80% of mice from the former group and 50% of mice from the latter group rejected the tumor implant and survived (Supplementary Materials Figure S1B). The antitumor responses elicited by the different treatments in the blood stream reflected the stimulation of cytotoxic responses within tumor microenvironment. In fact, mice receiving CTX injection in combination with anti-PDL1 and anti-PDL2 Abs showed a 3-fold increase in CD8^+^/Treg ratio (Figure 4E) and upregulated IFNγ and granzyme B expression in the tumor tissue as compared to the other treatments (Figure 4F). Instead, perforin expression was significantly upregulated in tumors from mice treated with CTX+anti-PDL1/2 as compared to all other groups, except for CTX + anti-PDL1 Ab (Figure 4F).

Mice responding to the combined chemo-immunotherapy treatment showed the upregulation of cytokine transcripts typical of a mixed Th1/Th2/Th17 profile (Supplementary Materials Figure S1H), which reflects the complexity and the variety of the leucocyte infiltrate composition observed. Indeed, we observed the preferential infiltration by Mo-MDSC, macrophages and eosinophils among myeloid cell subsets and by CD8^+^ and CD4^+^ T cells over Tregs into the tumor tissue of mice treated with CTX + PDL1/2 therapy as compared to the other treatments (Figure 4 G–H, Supplementary Materials Figure S1C–F). In addition, we observed an increase in central memory (Tcm) and effector memory (Tem) CD8^+^ T lymphocytes in the tumor-draining lymph nodes (LN) of animals treated with CTX and anti-PDL1 Abs as compared to each single treatment (Supplementary Materials Figure S1G). 

Overall, these results suggest that despite PDL1 blockade play a major role in dictating the therapeutic efficacy, concomitant blockade of PDL2 is required to achieve optimal antitumor immunity, which is orchestrated by the tight cooperation of myeloid and lymphoid cell subsets within the TME.

### 3.4. Immunogenic Chemotherapy Augments the Therapeutic Efficacy of PDL Blockade in Poorly Immunogenic Tumors

**Figure 5:**
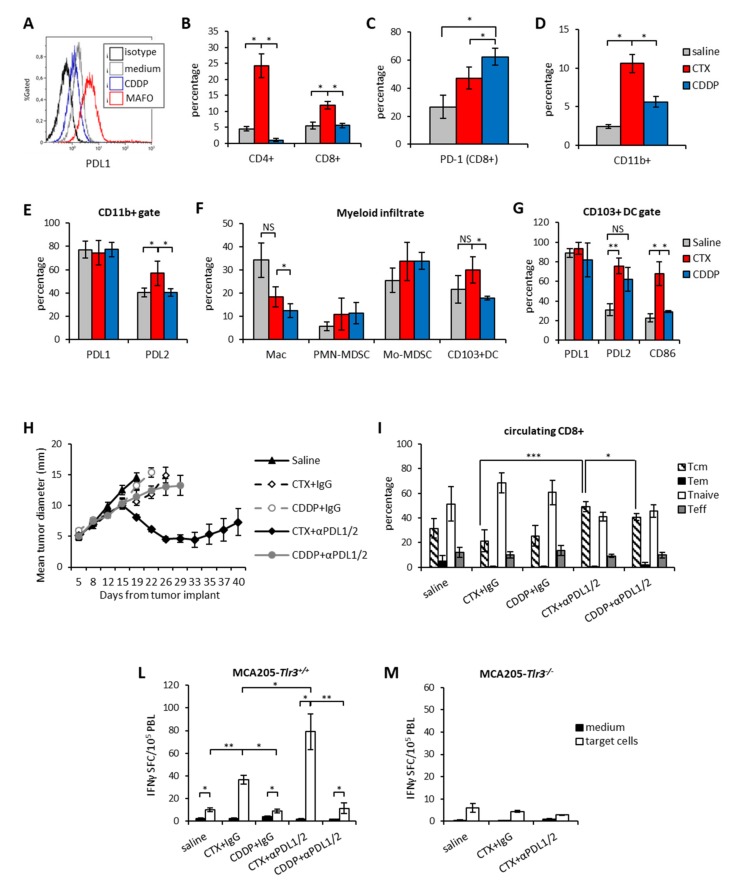
CTX, but not CDDP, pre-treatment synergizes with PDL1/2 blockade in vivo. **(A)** Surface expression of PDL1 on MCA205 tumor cells treated in vitro for 24 h with mafosfamide (15 µg/ml, MAFO) or cisplatin (CDDP) or with medium as control. One representative experiment out of two is shown. **(B)** Percentage of tumor-infiltrating CD4^+^ and CD8^+^ T lymphocytes 7 days after treatment with CTX (100 mg/kg) or CDDP (5 mg/kg) or Saline as control (*n* = 5). **(C)** Percentage of PD1 expression on tumor-infiltrating CD8^+^ T cells 7 days after treatment (*n* = 5). **(D)** Percentage of tumor infiltrating CD11b^+^ 7 days after treatment with CTX (100 mg/kg) or CDDP (5 mg/kg) or Saline as control (*n* = 6). **(E)** Percentage of PDL1 and PDL2 expression on tumor-infiltrating CD11b^+^ cells 7 days after the indicated treatments (*n* = 5). **(F)** Percentage of tumor-infiltrating DC in tumor beds collected 7 days after the indicated treatments and **(G)** Percentage of PDL1, PDL2 and CD86 expression tumor-infiltrating DC (*n* = 5). Data are expressed as mean ± SD. **(H)** Mean tumor size in mice treated with the indicated treatments (n = 6). **(I)** Relative abundance of circulating central memory (Tcm), effector memory (Tem), naive and effector (Teff) lymphocytes in mice on day 21 from chemotherapy administration (n = 6). **(L)** IFNγ-ELISpot from blood leucocytes (PBL) collected from mice implanted with MCA205-*Tlr3^+/+^* (*n* = 6) or **(M)** MCA205-*Tlr3^-/-^* and treated as indicated (*n* = 6). Assay was performed 4 days after the last Ab injection. Data are expressed as mean ± SEM. * *p* < 0.05; ** *p* < 0.01; *** *p* < 0.005. NS = non-significant.

The clinical efficacy of immune checkpoint inhibition has been positively associated with the intrinsic antigenicity of cancer cells, a major determinant of the magnitude of the antitumor immune responses [11,12]. We can thus speculate that the therapeutic synergy of PDL blockade and CTX in EG.7-OVA tumor model is influenced by the intrinsic immunogenicity of tumor cells which can be further augmented by immunogenic chemotherapy. In order to investigate whether inherent immunogenicity is required for the therapeutic success of combined chemo-immunotherapy we tested the same treatment modality in a less immunogenic experimental model. To this aim, we implanted female C57Bl/6 mice with MCA205 fibrosarcoma cells and selected two drugs with different immunogenic potential: CTX known to be an Immunogenic Cell Death (ICD)-inducer and cisplatin (CDDP) a non-ICD inducer [27]. We first tested basal PDL1 and PDL2 expression on MCA205 in vitro and observed that these cells express PDL1 (Figure 5A), but not PDL2 (Supplementary Materials Figure S2D). Interestingly, PDL1 expression was enhanced by treatment with mafosfamide (MAFO, the in vitro active analogue of CTX) while it was not affected by treatment with CDDP (Figure 5A). 

In line with previous reports [28], PDL1 upregulation by MAFO was dependent on IFNAR and TLR-3 signaling as it was abrogated in tumor cells where IFNAR1 was blocked by neutralizing Ab or in MCA*-Tlr3^-/-^* (Supplementary Materials Figure S2B,C). We then implanted subcutaneous MCA205 tumors in vivo to perform a phenotypical characterization of the immune infiltrate after treatment with either drug. Overall, CTX-treated tumors were enriched in both CD4^+^ and CD8^+^ T cells (Figure 5B) and CD11b+ myeloid cells as compared to saline or CDDP (Figure 5D). Expression of PD1 as well as of its cognate ligands were also analyzed on CD8^+^ and CD11b^+^ cells, respectively. CTX did not significantly increase the frequency of CD8^+^PD1^+^ cells that was instead increased by CDDP (Figure 5C). PDL1 was expressed by 80% of tumor infiltrating myeloid cells and not influenced by either drug treatment (Figure 5E). On the contrary, PDL2 was less expressed on CD11b^+^ cells than PDL1 in saline and CDDP-treated tumors (40–50%), but it was significantly enhanced by CTX (Figure 5E). Among myeloid cells, macrophages and the cross-presenting DC subset (CD103^+^ DCs) were enriched in CTX-treated tumors as compared to CDDP-treated tumors (Figure 5F). Of note, CTX treatment associated to PDL2 and costimulatory molecule upregulation in CD103^+^ DCs (Figure 5G). Based on the TME phenotypical analysis we administered the anti-PDL1/2 based chemo-immunotherapy to MCA205 tumor bearing mice in combination with either CTX or CDDP. As shown in Figure 5H, the mere chemotherapeutic treatment did not affect tumor growth independently of the drug type and the therapeutic effect of CDDP was not enhanced by combination with PDL1/2 blockade. On the contrary, significant tumor shrinkage was induced by anti-PDL1/2 in combination with CTX leading to complete tumor eradication in 30% of mice (Figure 5H and Supplementary Materials Figure S2F). Of note, in the absence of chemotherapy administration of anti-PDL1/2 Abs preceded by the injection of IFN-I (to increase PDL1 expression) did not exert any therapeutic effect as compared to control (Supplementary Materials Figure S2D–E) Although the frequency of circulating CD4^+^ and CD8^+^ lymphocytes was similar among the different groups of treatment (Supplementary Materials Figure S2G), mice receiving CTX and anti-PDL1/2 Abs displayed significantly more central memory CD8^+^ T cells (Tcm) than the other groups (Figure 5I). The analysis of tumor-specific immune responses showed that, as observed in the EG.7-OVA model, CTX treatment enhanced the number of tumor-reactive PBLs that further increased upon combination with anti-PDL1/2 (Figure 5L). On the contrary, CDDP therapy did not affect the antitumor immune response irrespective of checkpoint inhibition (Figure 5L). Since induction of a protective anticancer immune response to immunogenic chemotherapy requires type I IFNs signature through TLR3 signaling [29] we analyzed the role of this pathway in the response to PDL blockade. Treatment of mice bearing MCA205-*Tlr3^-/-^* with CTX or CTX + anti-PDL1/2 revealed that signaling through this receptor is crucially required for the achievement of a tumor-specific systemic immunity. In fact, in these mice treatment with either CTX or combined CTX + anti-PDL did not induce any increase in circulating tumor specific CD8^+^ T cells meaning that TLR-3 signaling in tumor cells is crucially required for the immunostimulatory effect of CTX (Figure 5M). These results show that the therapeutic efficacy of our chemo-immunotherapy protocol correlate with enhanced tumor-specific immunity and increase of circulating central memory frequencies also in a poorly immunogenic tumor model. The comparison between CTX and CDDP suggests that immunomodulatory properties of chemotherapy play a role in dictating the success of the combination [15]. 

### 3.5. Chemotherapy-Induced Vaccination only Partially Explains the Therapeutic Synergism between Immunogenic Chemotherapy and PDL Blockade

**Figure 6:**
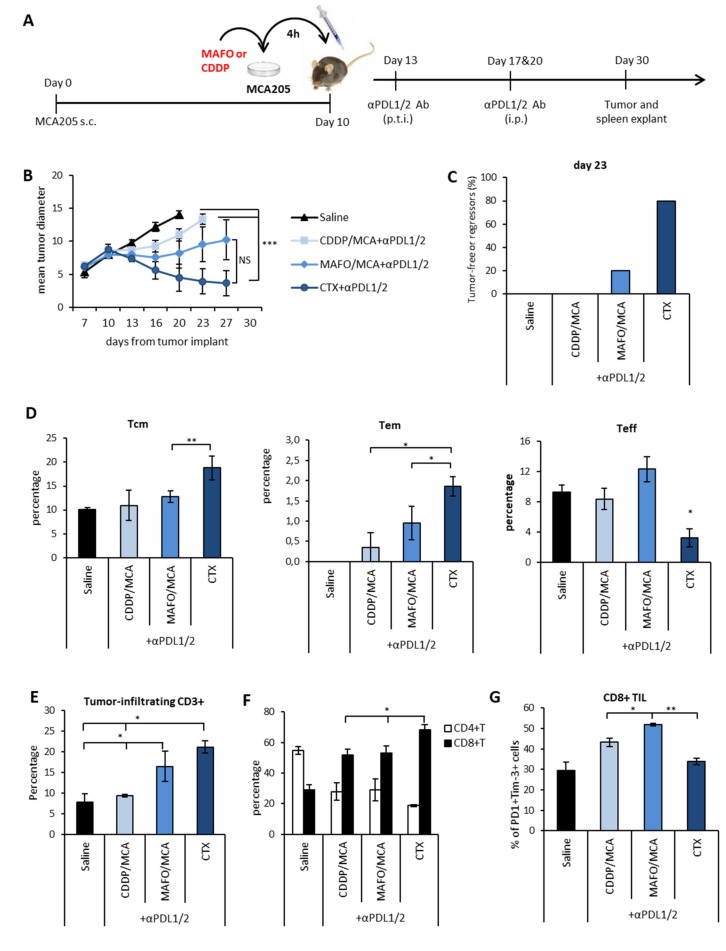
Vaccine-like effect of chemotherapy was necessary, but not sufficient for synergism with PDL blockade. **(A)** Schematic representation of the experimental design. MCA205 tumor cells were forced to die by in vitro incubation with either CDDP or MAFO for 4 h, then washed and injected s.c. at the tumor site followed by three anti-PDL1/2 injections as indicated. **(B)** Mean tumor size ± SEM in mice treated with the indicated treatments (*n* = 5). **(C)** Percentage of tumor-free or regressing tumors in each experimental group on day 23 from treatment initiation. One representative experiment out of two with similar results is shown. **(D)** Frequency of central memory (Tcm), effector memory (Tem) and effector (Teff) CD3^+^CD8^+^ lymphocytes in spleens 20 days after treatment initiation (*n* = 5). **(E)** Percentage of CD3^+^ TIL and **(F)** relative abundance of CD4^+^ and CD8^+^ TIL (*n* = 5). **(G)** Expression of PD1 and Tim-3 on CD3^+^CD8^+^ TIL. Data are expressed as mean percentage ± SEM (*n* = 5). * *p* < 0.05; ** *p* < 0.01; *** *p* < 0.005. NS = non-significant.

Given the synergistic effect of CTX, but not of CDDP, pre-treatment on anti-PDL therapy and the absence of tumor-specific CD8^+^ T cells in mice implanted with MCA205-*Tlr-3^-/-^* treated with CTX + anti-PDL1/2 Ab, we hypothesized that the induction of ICD by CTX could be the key mechanisms underlying the synergisms between chemotherapy and PDL blockade. To investigate this issue, MCA205 tumor cells were incubated with either CDDP or MAFO for 4 h to induce the necrotic/apoptotic program and the concurrent immunogenic potential (Supplementary Materials Figure S3). Drug-killed cells were then injected peritumorally in MCA205 tumor bearing mice to mimic the cell death that occurs in vivo after chemotherapy administration. The MAFO/MCA or CDDP/MCA injections were followed by anti-PDLs administration (Figure 6A). 

One group of mice was exposed to the on target and off target effects of chemotherapy by receiving a systemic administration of CTX i.p. followed by anti-PDLs. At early time points from vaccine or drug administration (i.e., day 13–16), a stabilization of tumor growth occurred in all treatment groups as compared to control group (saline-treated). At later time-points, MAFO or CDDP-killed tumor cells were no longer effective in controlling tumor growth. Interestingly, immunogenic MAFO-killed tumor cells were slightly more effective than CDDP-killed cells in restricting tumor growth following anti-PDLs (Figure 6B). In vivo treatment with CTX + anti-PDL1/2 confirmed its superior therapeutic efficacy (Figure 6B–C) and was accompanied by a significant increase in memory subsets (Tcm and Tem) as compared to effector subsets in the spleen (Figure 6D), suggesting that the therapeutic effect of CTX in combination with PDL blockade can be ascribed only in part to the induction of ICD. The analysis of tumor infiltrating lymphocytes showed an increase in CD8^+^ T cells and a concomitant decrease in CD4^+^ T cells, positively correlating with the therapeutic outcome (Figure 6E–F). Furthermore, increased frequencies of tumor infiltrating PD1^+^Tim-3^+^CD8^+^ T cells were observed in the MAFO/MCA group versus group treated with CDDP/MCA or with systemic CTX, indicating that immunogenic vaccine exacerbates T cell stimulation of active tumor control (Figure 6G). These results suggest that the immunogenic properties of CTX contribute to the therapeutic success of the combined chemo-immunotherapy treatment. Nevertheless, additional off-target immunomodulating properties accompanying the in vivo administration of the drug are required to achieve the best therapeutic outcome. 

## 4. Discussion

In the present study, we show that the therapeutic efficacy of anti-PDL1 and anti-PDL2 Abs can be significantly enhanced by the timely administration of immunogenic chemotherapy and that the effectiveness of this combination is proportional to the inherent immunogenicity of the tumor. It is well established that immunogenic tumors respond to ICIs better than non-immunogenic tumors and that the intensity of PDL1 and MHC class I expression dictates the efficacy of immunotherapy [30]. In our setting, treatment of mice bearing an immunogenic tumor (EG.7-OVA) with anti-PDL1 and anti-PDL2 Abs increased the frequency of circulating IFNγ-secreting CD8^+^ T cells but had scarce effect on tumor-specific immunity and no therapeutic advantage over placebo. However, treatment of the same tumor with a single dose of CTX followed by anti-PDL1 and anti-PDL2 Abs cured 60% of mice with no tumor-relapse and induced the establishment of local and systemic long-lasting tumor specific immunity. Cyclophosphamide is a cytotoxic drug with well-known immune-activating properties [15]. These include: i) the stimulation of a cytokine storm [31,32], ii) the induction of immunogenic cell death (ICD), thus converting dying tumor cells into an endogenous vaccine [16], iii) the stimulation of lymphocyte proliferation and activation [32], iv) the transient reduction of suppressor cells [33] and v) the polarization of T helper 1 (Th1) and Th17 responses [34]. All these effects occur with a precise timing and are dose and schedule dependent. In this respect, we can speculate that the administration of anti-PDL1/2 Abs soon after CTX injection benefits from all these “off target” mechanisms and boosts tumor-specific immune responses before the immune-stimulating effect of the drug is exhausted. Interestingly, although PDL1 blockade appears to play the major role in the combined chemo-immunotherapy, optimal stimulation of tumor-specific cytotoxic immunity and long-term disease control requires concomitant PDL2 blockade. Of note, PDL2 was independently associated with clinical response in anti-PD1-treated head and neck cancer patients, indicating that presence or absence of PDL2 expression may play a role in the response to ICI-based immunotherapy [35]. In a poorly immunogenic tumor model (MCA205), the type of chemotherapy used (CTX versus CDDP) dictates the therapeutic outcome of the combination both in terms of mice survival and of antitumor immunity induced. The synergistic effect observed is independent of the mere PDL1 expression by tumour cells as combination of anti-PDL1/2 Abs with a PDL1 inducer (e.g. IFN-I) does not improve the outcome of therapy. Both CTX and CDDP induce PD1 upregulation in CD8^+^ T lymphocytes, thus suggesting an effect on adaptive immunity. However, these drugs display opposite capability to induce ICD and exert different effects on immune cells both in vitro and in vivo [15]. Accordingly, CTX and CDDP display different therapeutic outcomes in combination with anti-PDL Abs. It has been reported that ICD inducer drugs stimulate the release of nucleic acids, mostly RNA, from dying tumor cells. These molecules are sensed by endosomal TLR-3 in both viable tumor cells and tumor-infiltrating DC and activate complex signaling pathways, ultimately leading to interferon-stimulated gene (ISG) expression and type I IFN production [32]. Based on these results, type I IFN-I via TLR-3 activation has been proposed as a hallmark for successful chemotherapy response. Hence, tumors lacking TLR-3 or IFNAR fail to respond to immunogenic chemotherapy [29]. Also, PDL1 upregulation has been associated to IFN-I signaling and TLR-3 [25]. Our data suggest that PDL1 upregulation by MAFO, but not by CDDP, in tumor cells may reflect the sensing of IFN-inducing molecules released by dying tumor cells. In our model, antitumor responses associated to tumor regression were absent in mice implanted with *Tlr-3^-/-^* tumors, thus suggesting a role of this ling pathway in the synergism between CTX and anti-PDL Abs. In line with this observation, Takeda and collaborators showed that co-administration of TLR-3-specific RNA agonist and tumor vaccine in combination with anti-PDL1 antibody facilitated tumor regression in mice [36], implying that signaling through this receptor is necessary for the success of the combination. Although CDDP does not induce ICD, it has been reported to upregulate MHC class I expression and to stimulate the effector functions of CD4^+^ and CD8^+^ lymphocytes in preclinical and clinical settings [37]. In the B16 murine melanoma model, pre-treatment with a single dose of CDDP (5 mg/kg) enhanced the efficacy of a cytokine-induced killer (CIK) infusion. This effect was attributed to the augmented homing ability of exogenous and endogenous effector cells and to the induced modulation of the myeloid cells [38]. Indeed, in the MCA205 model, mice treated with CDDP showed the enhanced expression of PD1 by tumor-infiltrating CD8^+^ T lymphocytes, but no effect on tumor infiltration by T cells or myeloid cells was observed. Our data demonstrate that despite being a necessary phenomenon, the vaccine-like effect induced by ICD is not sufficient per se to justify the synergism between CTX and ICIs. In fact, the injection of immunogenic tumor apoptotic bodies (MAFO/MCA) followed by ICIs did not recapitulate the therapeutic outcome observed with systemic CTX administration. Mice injected with MAFO/MCA displayed comparable T cell infiltrates than mice injected with CTX, but the former had more terminally differentiated effector CD8^+^ T cells (PD1^+^Tim-3^+^). These are efficient killers in acute immune reactions but have poor proliferative capability and long-term survival [39]. In our vision, the intratumoral recruitment of myeloid cells and in particular of cross-presenting DCs (CD103^+^ DCs) plays a key role in the synergisms. These cells accumulated in the tumor bed of mice treated with CTX, but not with CDDP, where they became competent to prime CD8^+^ T cells, in line with our previous observation [16]. Among antigen presenting cells, CD103^+^ DCs are uniquely capable of transporting antigens to the tumor-draining LN and prime CD8^+^ T cells [37,38,39]. Their selective ablation massively impairs T cell mediated tumor regression [40]. In a model of vaccination, adjuvant-driven recruitment of CD103^+^DCs was required to induce significant memory CTL responses to ovalbumin [41]. Expression of inhibitory molecules, including PDL1 and PDL2, by DCs mitigates their stimulatory ability in the TME, thus limiting CD8^+^ T-mediated responses [42]. In the tumor models analyzed, CD103^+^DCs primarily expressed PDL2 among myeloid cells and, in MCA205 fibrosarcoma, PDL2 was further upregulated by CTX. Thus, we can speculate that the addition of anti-PDL2 Ab to anti-PDL1 therapy soon after immunogenic chemotherapy maximizes the ability of CD103^+^DCs to cross-present tumor antigens by shielding two non-redundant immunoinhibitory signals, ultimately leading to an efficient and long-lasting CD8^+^T cell stimulation. Along these lines, Mayoux and colleagues have recently shown that blocking PDL1 on DCs allow a better stimulation of T cell priming with positive immunological and likely clinical consequences for checkpoint blockade therapy in cancer patients [43]. In addition to CD103^+^ DCs, intratumoral recruitment of eosinophils may contribute to tumor rejection through both direct cytotoxicity towards tumor cells and by secreting chemokines capable of attracting CD8^+^ T cells as recently reported [20,44,45]. The increase in tumor-infiltrating eosinophils in mice exposed to CTX/anti-PDL1/2 correlates with intratumoral expression of IL-33 which can both recruit and activate these cells [46]. Importantly, eosinophils are emerging as relevant immune biomarkers for response to ICIs in cancer patients, correlating with better clinical outcome [47]. In conclusion, the composition of the tumor-associated myeloid compartment plays a key role in tumor response to anti-PDL blockade. Immunogenic chemotherapy capable of maximizing myeloid cell tumor infiltration and activation may represent an ideal candidate for combination with ICIs. Overall, these studies may contribute to the design of rationale-driven clinical studies of chemo-immunotherapy for cancer patients.

## Figures and Tables

**Table 1:** List of forward and reverse primers used for real-time quantitative PCR.

Gene	Forward and Reverse Primers (5′–3′)	NCBI Accession Number	Amplicon Size (Base Pairs)
*IFN-* *γ*	TCAAGTGGCATAGATGTGGAAGAA TGGCTCTGCAGGATTTTCATG	NM_008337.4	92 bp
*IL-2*	CCTGAGCAGGATGGAGAATTACA TCCAGAACATGCCGCAGAG	NM_008366.3	141 bp
*IL-4*	ACAGGAGAAGGGACGCCAT GAAGCCCTACAGACGAGCTCA	NM_021283.2	95 bp
*IL-10*	GGTTGCCAAGCCTTATCGGA ACCTGCTCCACTGCCTTGCT	NM_010548.2	191 bp
*IL-12*	GGAAGCACGGCAGCAGAATA AACTTGAGGGAGAAGTAGGAATGG	NM_001303244.1	180 bp
*IL-13*	AGACCAGACTCCCCTGTGCA TGGGTCCTGTAGATGGCATTG	NM_008355.3	123 bp
*IL-17*	GCTCCAGAAGGCCCTCAGA AGCTTTCCCTCCGCATTGA	NM_010552.3	142 bp
*IL-33*	GGGCTCACTGCAGGA GGACCAGGGCTTCGC	NM_ 001164724.2	147 bp
*TGF-* *β1*	TGACGTCACTGGAGTTGTACGG GGTTCATGTCATGGATGGTGC	NM_ 011577.2	170 bp
*Prf1*	GTGTCGCATGTACAG TGTGGTAAGCATGCT	NM_ 011073.3	116 bp
*Gzmb*	GATCGGGAGTGTGAGTCCTAC GAAAGCACGTGGAGGTGAAC	NM_013542.2	183 bp
*TLR-3*	TTGCGTTGCGAAGTGAAGAA CAGTTGGGCGTTGTTCAAGA	NM_126166.5	149 bp
*HPRT*	CTGGTGAAAAGGACCTCTCG TGAAGTACTCATTATAGTCAAGGGCA	NM_013556.2	109 bp

## Supplementary Materials

The following are available online at https://www.mdpi.com/2073-4409/9/4/940/s1, Figure S1: Analysis of immune correlates in mice treated with CTX + anti-PDL; Figure S2: Role of PDL1 modulation in the therapeutic outcome of combined chemo-immunotherapy; Figure S3: Different immunogenic potential of CDDP and CTX.
