# Supplementary material for: Tumor-Intrinsic or Drug-Induced Immunogenicity Dictates the Therapeutic Success of the PD1/PDL Axis Blockade
Source: Cells. 2020 Apr 10;9(4):940. doi: 10.3390/cells9040940 (PMC7226952; doi:10.3390/cells9040940)
Supplement: Supplementary file 1 [file cells-09-00940-s001.zip › Supplementary figure legends.docx]

**Supplementary figure legends.**

**Supplementary Figure 1.** (A) Frequency of IFNγ secreting blood leucocytes (PBL) collected from long-term survivors on day 100 from treatment initiation and stimulated with MHC class I-restricted OVA peptide (OVA p) or medium as control. Data are expressed as mean ± SEM. (B) Percentage of mice that rejected a challenge with live tumor cells. The number of mice per group is indicated in brackets. (C) Regulatory T cell/CD3^+^ ratio in spleen and tumor of mice treated as indicated analyzed 14 days after treatment initiation (n=4). Data are expressed as mean fold change ± SD and normalized to saline-treated group. (D) Percentage of CD3^+^CD8^+^ and of (E) CD3^+^CD4^+^ tumor-infiltrating leucocytes (TIL) on day 14 from treatment initiation (gate on viable cells) (n=4). (F) Percentage of myeloid cell subsets in the tumor mass analyzed on day 14 from treatment initiation after gating on CD11b^+^ cells. Data are expressed as mean ± SD (n=4). (G) Percentage of Tcm and Tem CD8+T cells in the LN of mice treated as indicated (n=4). (H) Heatmap view of gene expression upon in vivo treatment with the indicated experimental conditions. Tumor masses were explanted on day 14 after treatment initiation (n=4). Data are expressed as Log10FC, logarithmic scale of mean values, normalized for each indicated gene. * p<0.05; ** p<0.01; *** p<0.005; NS=non-significant.

**Supplementary Figure 2.** (A) TLR-3 expression on native MCA205 tumor cells and on their *Tlr3^-/-^* counterpart. Data have been normalized vs HPRT housekeeping and are expressed as mean expression ± SEM. (B) Surface expression of PDL1 on MCA205-*Tlr3^+/+^* tumor cells after gating on viable cells (NiR-neg) 24h after incubation with MAFO (10µM) in the presence of anti-IFNAR1 Ab or Control IgG. (C) Surface expression of PDL1 on MCA205-*Tlr3^-/-^* 24h after incubation with MAFO (10µM) or with medium as contol. Histogram plots represent PDL1 expression after gating on viable cells (Nir-neg). One representative experiment out of two with similar results is shown. (D) Surface expression of PDL1 and PDL2 on MCA205 tumor cells after gating on viable cells (NiR-neg) 24h after incubation with IFN-I (1000U) or mock. Untreated tumor cells (medium) or treated tumors cells stained with isotype Ab are shown as controls. (E) Percentage of surviving mice treated with IFN-I followed, 24h apart, by three injections of anti-PDL1 and anti-PDL2 Abs according to the schedule described in Materials and methods section (n=5). (F) Percentage of surviving mice treated as indicated (n=6). (G) Percentage of CD3^+^CD4^+^ and CD3^+^CD8^+^ in the blood of treated mice collected on day 20 after treatment initiation. Data are expressed mean values ± SEM (n=6). **-p<0.01

**Supplementary Figure 3.** MCA205 tumor cells were incubated for 4h with CDDP (6 µM) of MAFO (10 µM) dissolved in culture medium. Treated tumor cells were then extensively centrifuged and plated again for additional 24h to verify the release of immuno-activating signals by dying tumor cells. (A) Representative pictures from culture wells of MCA205 tumor cells incubated with the indicated drugs of with medium (NT) as control. (B) Morphological parameters of MCA205 treated as indicated. (C) Mean fluorescence intensity (MFI) of the indicated surface markers after 24 h of culture with supernatants from MAFO-treated or CDDP-treated MCA205. (D) Representative histogram plots of CD86 and CD40 expression on DC after colture with the indicated supernatants. One representative experiment out of three with similar results is shown. * p<0.05; ** p<0.01; *** p<0.005.
