# Supplementary figures and images for: Tumor-Intrinsic or Drug-Induced Immunogenicity Dictates the Therapeutic Success of the PD1/PDL Axis Blockade
Source: Cells. 2020 Apr 10;9(4):940. doi: 10.3390/cells9040940 (PMC7226952; doi:10.3390/cells9040940)

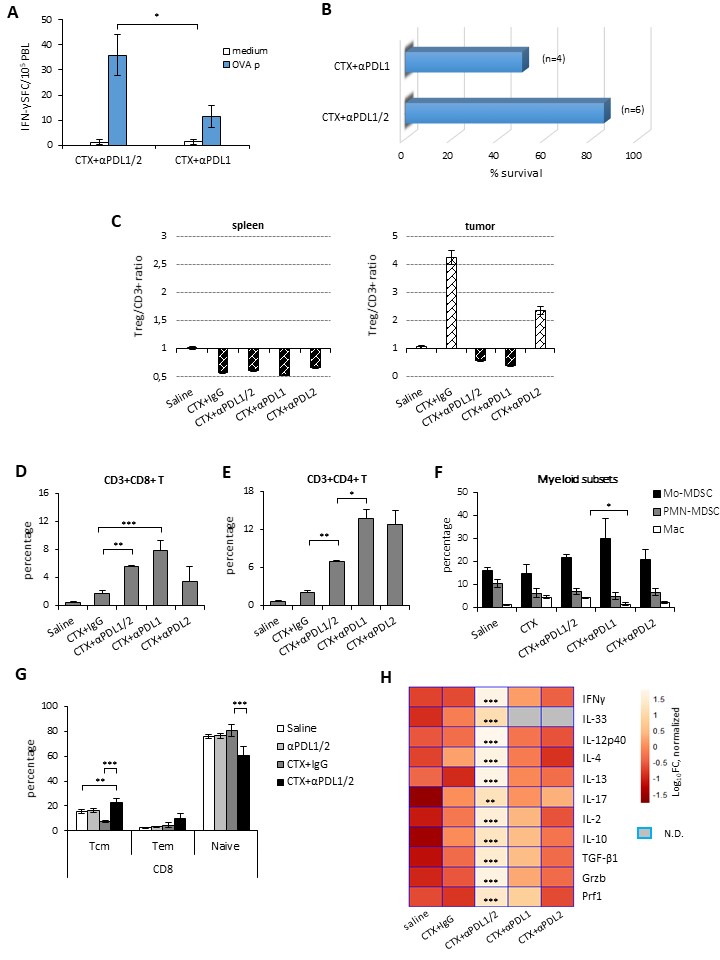

Supplement: Supplementary file 1 [file cells-09-00940-s001.zip › Diapositiva1.JPG]

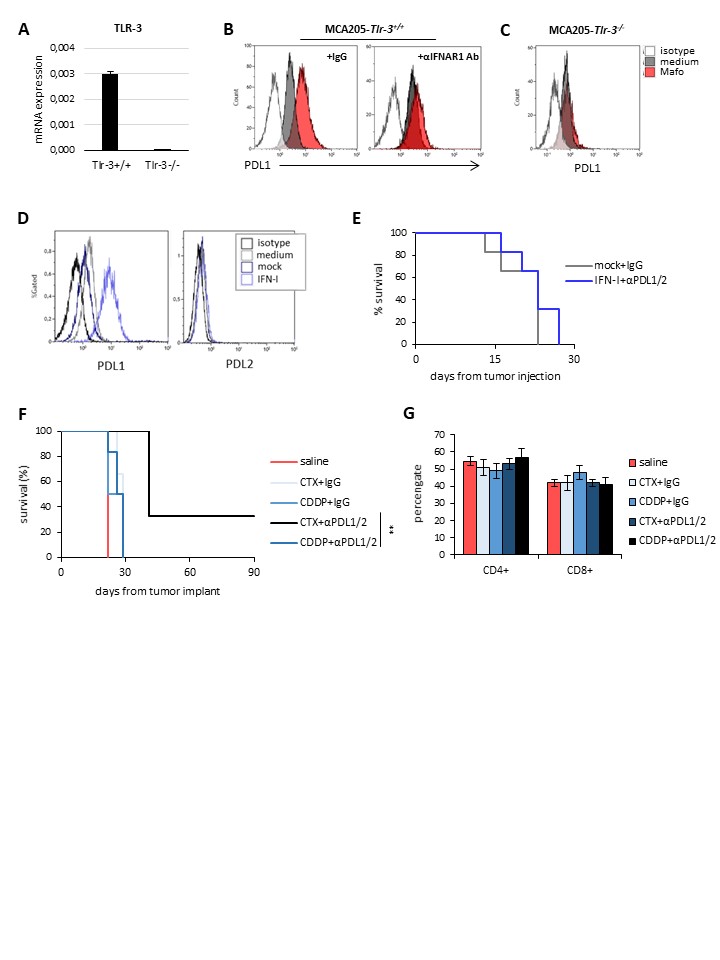

Supplement: Supplementary file 1 [file cells-09-00940-s001.zip › Diapositiva2.JPG]

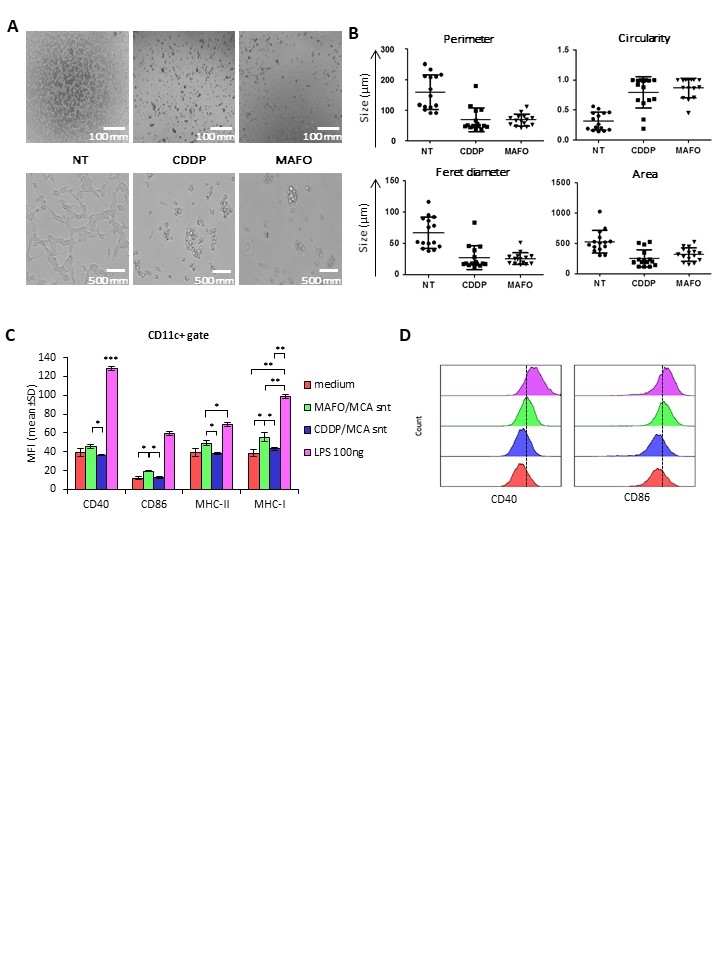

Supplement: Supplementary file 1 [file cells-09-00940-s001.zip › Diapositiva3.JPG]
